# Emergency medical service systems in Sri Lanka: problems of the past, challenges of the future

**DOI:** 10.1186/s12245-017-0127-x

**Published:** 2017-02-21

**Authors:** Kelum Wimalaratne, Jeong IL Lee, Kang Hyun Lee, Hee Young Lee, Jung Hun Lee, In Hye Kang

**Affiliations:** 1 Preliminary Care Unit, Base Hospital of Avissawella, Colombo, Sri Lanka; 2 Department of Emergency Medicine, Wonju College of Medicine, Yonsei University, Wonju, Republic of Korea; 3 Wonju College of Medicine, Yonsei University, 20, Ilsan-ro, Wonju, Gangwon Province 26426 Republic of Korea

**Keywords:** Emergency medical service, Prehospital care, Sri Lanka, Disaster, Emergency medicine

## Abstract

**Introduction:**

The concept of emergency medical services (EMS) is new to Sri Lanka. This article describes the development, delivery, and future ideas for EMS in Sri Lanka. Sri Lanka also faces frequent natural hazards that justify the establishment of an EMS service.

**Methodology:**

Data and information regarding emergency medical care in Sri Lanka were collected and reviewed from resources including websites and research papers.

**Results:**

Currently, there are no qualified emergency medical physicians in Sri Lanka. However, a specialist training program for emergency physicians was initiated in 2012. There is no formal system to train emergency medical technicians (EMTs). Sri Lankans usually use taxies or their private vehicles to get to the hospital in the case of an emergency. All of the hospitals have ambulances that they can use to transport patients between hospitals. Most hospitals have emergency treatment units. Those at larger hospitals tend to be better than those at smaller hospitals. Although there is a disaster management system, it is not focused on emergency medical needs.

**Discussion:**

Many aspects of the EMS system in Sri Lanka need improvement. To start, the emergency telephone number should cover the entire country. Training programs for EMTs should be conducted regularly. In addition, ambulances should be allocated for prehospital care. In the process of these developmental changes, public awareness programs are essential to improve the function of the EMS system.

**Conclusion:**

Despite many current shortcomings, Sri Lanka is capable of developing a successful EMS system.

## Background

**Fig. 1:**
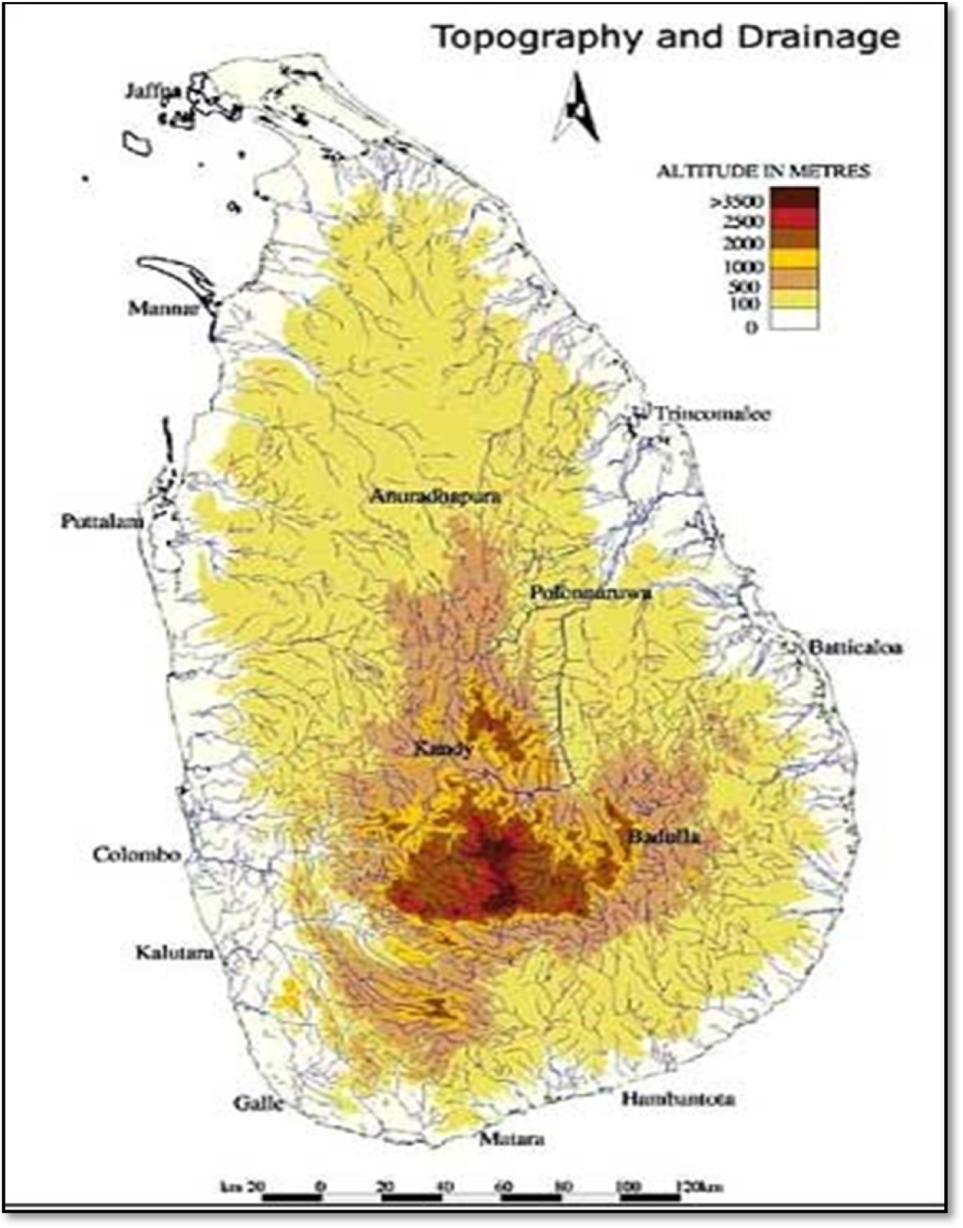
The geographic location and topographic character of Sri Lanka. These features have influence on the further necessity of EMS development in different districts. Accessed on 20 July 2014, at http://www.fao.org/ag/AGP/AGPC/doc/counprof/srilanka/srilanka.htm

Sri Lanka (formerly Ceylon) is a 65,620 km^2^ island in the Indian Ocean. It lies east of India’s southern tip, separated from the subcontinent by the Palk Strait. Its terrain is mostly flat with rolling plains, and mountains in the south-central interior. The core regions of the central highlands contain many complex topographical features including ridges, peaks, plateaus, basins, valleys, and escarpments (Fig. 1). There are 103 rivers in the Central Highlands that flow in a radial pattern toward the sea. Sri Lanka has warm weather year-round, which is moderated by the ocean winds and considerable moisture. It also has tropical forests and extensive biodiversity [1].

Sri Lanka has a documented history that spans over 3000 years. Buddhism was first introduced during the third century BC from India. This change led the country’s social and cultural development. Since the sixteenth century, the coastal areas have been ruled by the Portuguese, Dutch, and British. After 1815, the entire nation was under British colonial rule. Sri Lanka was granted its independence in 1948; however, the country remained a dominion of the British Empire. In 1972, Sri Lanka became a republic. Until today, Sri Lanka is a republic and unitary state, governed by a presidential system [2, 3].

After 26 years of civil war against the LTTE terrorists, the Sri Lankan government declared victory on May 18, 2009 [4]. Since then, Sri Lanka has continued to experience economic growth with 6–8% GDP growth and a GDP per capita (PPP) of approximately 7000 USD [5, 6].

Sri Lanka is the 57th most populated nation in the world, with 21 million people and an annual population growth rate of 0.73%. The country has a high literacy level (92.5%), a high life expectancy (75 years), and a low infant mortality rate (14 per 1000 live births) [7, 8]. These figures are comparable to those of developed countries [7, 8]. By the end of 2011, Sri Lanka had 16,384 qualified doctors in the state health sector, or one doctor for every 1274 persons [9]. By the end of 2012, there were 592 government hospitals with a total of 69,731 beds. This amounts to approximately 3.3 beds/1000 persons, not including private hospital beds.

Trauma is the leading cause of hospitalization in Sri Lanka. Approximately 600,000 trauma patients are hospitalized each year in government hospitals. Most of the moderate to severe trauma cases result from motor vehicle accidents. There are about 150 accidents reported daily, with 5–6 subsequent deaths [10, 11]. The other leading causes of injury-related deaths include suicide, collective violence, road injuries, drowning, and falls [12]. Most self-inflicted deaths are the result of poisoning. Poisoning accounts for ~80,000 hospitalizations and >3000 deaths per year [13, 14].

During the past half-century, the proportion of deaths due to circulatory diseases including ischemic heart disease and stroke has increased from 3 to 24%. The mortality rate from noncommunicable diseases (NCDs) is currently 20–50% higher in Sri Lanka than it is in developed countries. About 65% of deaths in Sri Lanka are due to NCDs. These NCDs result from sedentary lifestyles, smoking, unhealthy diets, and excessive liquor consumption [15].

**Fig. 2:**
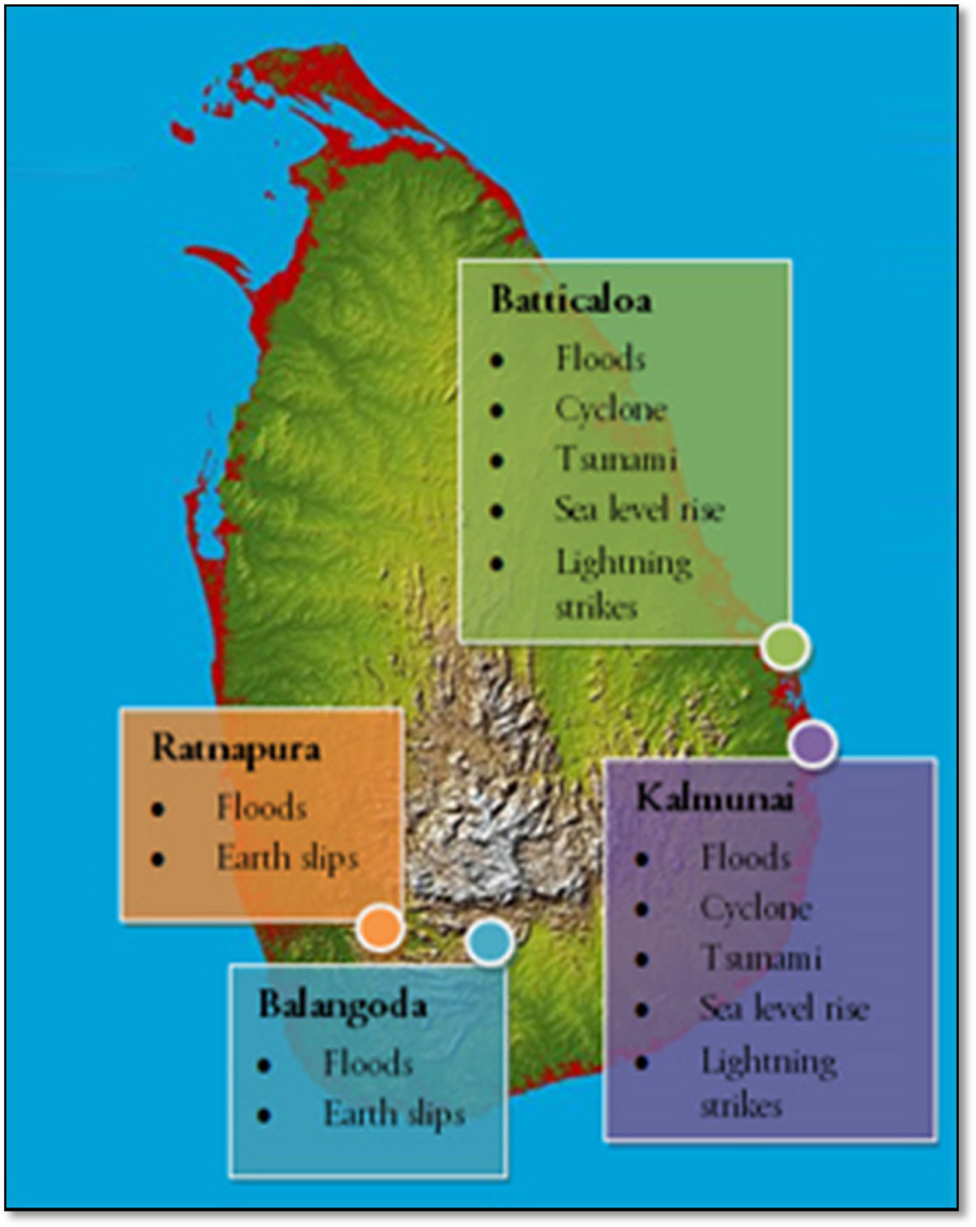
Floods, cyclone, tsunami, sea level rise, lightning strikes, and earth slips occurred at this geographic location of Sri Lanka. Accessed on 20 July 2014, at https://en.wikipedia.org/wiki/Geography_of_Sri_Lanka

The major natural hazards in Sri Lanka include floods, landslides, cyclones, droughts, wind storms, coastal erosion, tsunamis, sea surges, and rises in the sea level rise. Other more local hazards include lightning strikes, epidemics, high winds, fires, and wild elephant attacks (Fig. 2). These natural disasters are not only life threatening, but also can cause enormous damage and destruction of property [14, 16]. For example, the tsunami on December 26, 2004 was the biggest natural disaster that Sri Lanka has ever faced. Almost two thirds of the country’s coastal belt was vulnerable to the storm. Over 35,000 people were killed, approximately 5000 people went missing, and ~100,000 houses were completely destroyed. In total, 260,967 families and 1.3 million people were affected in some way by the tsunami. In addition, approximately 65% of the country’s fishing fleet (of 29,700 boats) was either damaged or destroyed [14, 17].

After this incident, both local and international health organizations became interested in establishing an emergency medical service in Sri Lanka. With foreign help, the Prehospital Care Committee was established as part of the Trauma Secretariat of the Sri Lanka Health Ministry. Subsequently, international medical teams from other countries collaborated with the Sri Lanka Health Ministry to develop prehospital care systems [10]. Unfortunately, given new laws and situation around the war including night-time military curfew, this effort was only successful in the city of Jaffna [18].

The purpose of this article is to review the current status of the prehospital care system in Sri Lanka and the efforts taken to improve it.

### Methodology

Data and descriptions regarding the prehospital system in Sri Lanka were gathered from relevant web sites and research papers. Information was also obtained directly from medical doctors and nurses who work in emergency treatment units and intensive care units in Sri Lanka. Finally, we also used the authors’ experiences working in IDP (Internally Displaced People) camps (following the tsunami disaster and war against terrorists), and in emergency medical care units (in a district and base hospital) as sources of information.

## Results

### Man power

Currently, there are no emergency medical physicians in Sri Lanka [19]. Instead, general physicians, orthopedic surgeons, and anesthesiologists supervise emergency treatment units. Almost all of the emergency medical technicians (EMTs) are only qualified in EMT level 1. Their service is also limited to a few areas in the country. Finally, there is no established system to recruit EMT-trained policemen and teachers [20]. Usually, community responders are responsible for prehospital care. Fortunately, these individuals are well aware of time-sensitive situations and make efforts to transport patients to the hospital as quickly as possible. Although they are commended for their volunteerism, however, they have very limited knowledge and experience in safe patient care and transport [21]. There are no properly trained emergency medical dispatchers in Sri Lanka [20].

### Training

In 2011, the Post Graduate Institute of Medicine at the University of Colombo established a multidisciplinary specialty board in emergency medicine to facilitate a specialist training program. With the help of members from the Australasian College of Emergency Physicians, they developed a comprehensive 6-year training curriculum that was approved by the Health Ministry in Sri Lanka. After a selection examination, the first batch of emergency medicine trainees was recruited in 2012 [19]. The Prehospital Care Sub-Committee has established minimum standards for EMT education that are comparable to those of the American National Registry of EMTs, the Australian Registry of EMTs, and the UK College of Emergency Medicine standards [10]. There are four levels (1–4) of EMT training. EMT level 1 included first aid and advanced first aid (basic rescue, oxygen use, CPR, splinting) training. These individuals are equivalent to first responders in the USA. EMT level 2 training standards are similar to those of EMT basic training in the USA. Level 2 training includes 120–160 h of classroom and clinical education about oral and nasal airways, automatic external defibrillators, spinal immobilization, and vital sign measurement. The training programs for EMT levels 3 and 4 are equivalent to those of EMT intermediate and EMT paramedic in the USA, respectively [10]. However, EMTs are not yet being trained to levels 3 and 4 in Sri Lanka. After the tsunami in 2004, Medical Teams International (under the supervision of the Ministry of Health and Nutrition) trained more than 2500 people, mostly to EMT level 1. These trainees included firemen, policemen, and teachers. Besides this effort, there is no ongoing training program for EMTs in Sri Lanka [20]. NGOs hold some training programs for first responders. However, these tend to be infrequent and poor in quality. In November of 2011, the Sri Lankan Society of Critical Care and Emergency Medicine (SSCCEM) initiated an “Emergency Life Care” training program based on the principles of the “Emergency Life Support” course in Australia [19]. In February 2011, Med 1 (PVT) Limited opened the first internationally certified training institute in Sri Lanka. This program is authorized to issue certifications in cardio-pulmonary resuscitation (CPR), advanced cardiovascular life support (ACLS), pediatric advanced life support (PALS), and first aid [22]. Medical doctors and nurses who work primarily in emergency treatment units, intensive care units, and surgical theaters often become involve in these programs to refresh their knowledge and skills. There is no proper training program for emergency medical dispatchers (EMD) in Sri Lanka. So far, there is only a 3-h introductory course on communicating and dispatching procedures [20].

### Communication

The emergency telephone number for EMS, reserved by the Telecommunications Regulatory Commission in Sri Lanka, is 1-1-0. This service can be accessed through any land or mobile phone [10, 18, 20].

Unfortunately, however, this number is not widely used, for several reasons. Some of the challenges include a lack of professional dispatchers, and service that is limited to very small areas. Some EMS systems can only respond to calls within a 5-km radius from their center. Furthermore, the system experiences frequent technical errors and generally has a lack of public awareness. Finally, it is only available within the eight EMS systems mentioned above. Even within the areas that it serves, less than 25% of people know the three-digit emergency number. This problem can mostly be attributed to a lack of public awareness campaigns [20]. There are major issues with communication when it comes to prehospital care. Most of the time, community responders bring the patient to a receiving center without any prior communication [20]. Very rarely, a citizen or traffic police member will call a receiving center about a patient in transit. In these situations, the “telephone-call exchange unit” of a particular hospital receives the call. Unfortunately, however, these massages are often not properly transferred to a responsible person in the emergency treatment unit. Alternatively, the patient may end up being taken to a different hospital altogether.

### Transportation

The International Center on Emergency Techniques (ICET) in The Netherlands upgraded some of the fire brigades in Sri Lanka by providing ambulances and rapid intervention vehicles (RIV). In addition, the Regional Health Department in Jaffna (the Northern-most Sri Lankan city) owns two boat ambulances to transport patients from the small, surrounding islands [20]. All of these resources are only available to the country’s eight EMS systems. Most people are transported to the hospital via three-wheeled taxies or small, private cars [21]. These three-wheelers are very small vehicles that only have room for two to three passengers. Patients are usually accompanied by at least two other people. Therefore, small taxies are not ideal for traveling patients, and can actually increase the risk of secondary injuries, especially in those with spinal cord injuries, or limb fractures, for example. Still, taxies are somewhat advantageous because they are readily available and can easily bypass heavy traffic. There are a few private ambulance services in the main cities that charge a fee for their service. Usually, affluent families use these services to transport their chronically ill or elderly parents to and from the hospital. Fortunately, there are nation-wide ambulances that are available for transferring patients between hospitals. Gradually, these ambulances are transforming from a simple transport vehicle to a mobile treatment and stabilization unit [10].

### Facilities and critical care units

Almost every hospital in the country has an emergency treatment unit. There are three levels of emergency units in Sri Lanka:

1. Accident Service in National Hospital.
2. Preliminary Care Units.
3. Emergency Treatment Units.

The Accident Service in the National Hospital (in Colombo) is the largest and best trauma center in Sri Lanka. It was established in 1992 as the first triage unit in Sri Lanka [23]. The center consists of seven operating theaters, two intensive care units, and two observation wards. There is a consultant surgeon on the premises at all times. Several large city hospitals have preliminary care units (PCU), with variable services and facilities. For the most part, they all have “emergency treatment beds” for medical emergencies, a “dressing area,” or a mini theater for surgical emergencies and an “observation ward.” The two largest PCUs belong to the Base Hospitals in Avissawella and Panadura. These hospitals were constructed with the financial assistance from the Korea Foundation for International Healthcare (KOFIH) and the Western Provincial Council of Sri Lanka. KOFIH continues to provide assistance by supplying medical equipment to Avissawella PCU, training the medical staff, and offering foreign training programs in Korea [24].

Almost all district level hospitals have emergency treatment units (ETUs). Usually these are small rooms with one to two beds and basic facilities such as a nebulizer, an ECG machine, and a glucometer. Large city hospitals have more advanced ETUs, but they are still very small compared to PCUs [25].

### Disaster management

In accordance with the Sri Lanka Disaster Management Act No. 13 of 2005, the National Council for Disaster Management established The Disaster Management Center (DMC). The Sri Lanka Disaster Management Act is the main legal document for disaster management in Sri Lanka that was passed by parliament in May 2005.

The DMC coordinates and collaborates with national and local administrative authorities, the armed forces and police, and international and local NGOs during disaster situations. During a disaster, it mainly focuses on relief services including food, clothes and shelter, rather than on emergency medical needs [26, 27].

## Discussion

An effective EMS system is imperative in Sri Lanka, given the country’s geography, demographics, and frequent natural disasters. However, Sri Lanka has a long way to go to offer integrated, efficient, and functional prehospital care.

First, the country needs a centralized communication center with properly trained dispatchers. Such a resource would communicate with various emergency services including the fire brigades and police department to offer support in the setting of civilian EMS needs, mass casualties, and disaster response activities.

The Trauma Secretariat must expand (and therefore strengthen) EMT training beyond establishing standards and publishing training materials (textbooks and DVDs). It is also necessary to establish a nationwide paramedic profession with formal training and qualification.

The Ministry of Health should consider allocating ambulances with a proper monitoring system, potentially by collaborating with the private ambulance services that already exist.

It is very important that public awareness campaigns about EMS access are conducted nationally. Given the high rates of volunteerism in Sri Lanka, enhanced public education regarding first aid and CPR may also help.

Sri Lanka is one of the few countries in the world that provides free healthcare. Therefore, higher officials in the Ministry of Health and in the government should consider allocating more funds to EMS services. However, as a developing country, this is easier said than done. Sri Lanka needs the financial support from the international community to make these efforts successful.

## Conclusions

Although the EMS services in Sri Lanka are still very immature, the country has the potential to establish an effective system. The country has an adequate infrastructure to develop the system under the Ministry of Health and has developed an international post-graduate specialist training program for medical doctors. The future of the EMS will depend on the recognition of emergency medicine as a primary specialty, public education campaigns on the role of the EMS personnel for medical safety, and investment in disaster management system such as medical infrastructure.
